# Risk Stratification of Pancreatic Cysts With Confocal Laser Endomicroscopy

**DOI:** 10.1016/j.gastha.2021.11.003

**Published:** 2022-02-03

**Authors:** Ritu R. Singh, Abhilash Perisetti, Kumar Pallav, Saurabh Chandan, Mariajose Rose De Leon, Neil R. Sharma

**Affiliations:** 1 Bloomberg School of Public Health, Johns Hopkins University, Baltimore, Maryland; 2 Department of Medicine, Indiana University School of Medicine, Fort Wayne, Indiana; 3 Department of Interventional Oncology and Surgical Endoscopy, Parkview Cancer Institute, Fort Wayne, Indiana; 4 Department of Gastroenterology, CHI Health, Creighton University Medical Center, Omaha, Nebraska

**Keywords:** Confocal, Endomicroscopy, Pancreatic Neoplasm, Intraductal Papillary Mucinous Neoplasm (IPMN)

## Abstract

In the modern era of high-quality cross-sectional imaging, pancreatic cysts (PCs) are a common finding. The prevalence of incidental PCs detected on cross-sectional abdominal imaging (such as CT scan) is 3%–14% which increases with age, up to 8% in those 70 years or older. Although PCs can be precursors of future pancreatic adenocarcinoma, imaging modalities such as CT scan, MRI, or endoscopic ultrasound with fine-needle aspiration (EUS-FNA) are suboptimal at risk stratifying the malignant potential of individual cysts. An inaccurate diagnosis could potentially overlook premalignant lesions, which can lead to missed lesions, lead to unnecessary surveillance, or cause significant long-term surgical morbidity from unwarranted removal of benign lesions. Although current guidelines recommend an EUS or MRI for surveillance, they lack the sensitivity to risk stratify and guide management decisions. Needle-based confocal laser endomicroscopy (nCLE) with EUS-FNA can be a superior diagnostic modality for PCs with sensitivity and accuracy exceeding 90%. Despite this, a significant challenge to the widespread use of nCLE is the lack of adequate exposure and training among gastroenterologists for the real-time interpretation of images. Better understanding, training, and familiarization with this novel technique and the imaging characteristics can overcome the limitations of nCLE use, improving clinical care of patients with PCs. Here, we aim to review the types of CLE in luminal and nonluminal gastrointestinal disorders with particular attention to the evaluation of PCs. Furthermore, we discuss the adverse events and safety of CLE.

## Introduction

Confocal laser endomicroscopy (CLE) (also known as an optical biopsy) is a novel diagnostic technique that utilizes a laser beam to obtain in vivo real-time pictures of the tissues that mimic histological images.^1^^,^^2^ CLE has been used in diagnosing luminal gastrointestinal (GI) disorders, such as Barrett's esophagus, colorectal neoplasms, and biliary strictures.^3^, ^4^, ^5^, ^6^ There is increasing evidence to support the diagnostic accuracy of CLE in pancreatic cysts (PCs) with better characterization of the cysts and, thus, indirect prediction of their malignant potential.^7^, ^8^, ^9^ These studies have shown exceptional accuracy of CLE in diagnosing the subtype of PCs (exceeding 90%), with high sensitivity and specificity.

As per recent literature, 2.5%–13% of patients undergoing abdominal imaging (CT or MRI) for unrelated indications have incidental detection of PC, depending on the imaging modality used and the mean age of the population studied.^10^^,^^11^ The prevalence increases with age and can be found in up to 40% of people older than 70 years.^12^^,^^13^ Although most PCs are incidental, they are not always benign. PC can be a harbinger of future pancreatic ductal adenocarcinoma (PDAC) with the 5- to 10-year risk of malignant transformation being 5%–8% in retrospective cohort studies. The highest risk is associated with intraductal papillary mucinous neoplasm (IPMN) greater than 1.5 cm in diameter.^14^^,^^15^ Consequently, the discovery of a PC imposes tremendous stress on the patient and the treating physician given the possibility of underlying pancreatic cancer or a precursor lesion.

Mortality in patients with PDAC (the third leading cause of cancer-related deaths) is increasing and attributed to late clinical presentation, lack of early detection strategies, complex pathobiology, and limited therapeutic options.^16^ There is no cost-effective surveillance strategy to identify this tumor at a curative stage. Most PDAC is thought to arise from PC, and IPMN is considered a precursor lesion in many cases. The increasing use of cross-sectional abdominal imaging has frequently resulted in the identification of PC. However, these imaging modalities have limited accuracy in characterizing PCs and in predicting their malignant potential. Current society guidelines endorse the use of endoscopic ultrasonography (EUS)–guided fine needle aspiration (FNA) for diagnosing suspicious or high-risk PC.^12^^,^^17^, ^18^, ^19^ However, the yield is limited by the paucity of cells in the aspirated fluid. The sensitivities of EUS morphology (approximately 50%) and cytology (about 60%), while superior to cross-sectional imaging alone, are suboptimal to make appropriate decisions whether to operate or continue surveillance for PC.^20^^,^^21^ Although a low carcinoembryonic antigen (CEA) (<5 ng/ml) can confidently rule out and high CEA (>800 ng/ml) can rule in mucinous cysts, the sensitivity is poor at approximately 50%.^20^^,^^21^ CLE can complement EUS in better characterization of the PC and simplify the surveillance scheme. Large-scale prospective multicenter studies are underway to confirm the encouraging role of CLE over guideline-directed imaging and biomarker-based diagnostic strategy in PC.

Here, we attempt to provide a comprehensive review of the role of CLE in the diagnosis and characterization of PC, associated challenges, and the limitations to its incorporation into routine clinical practice.

### Literature Search

**Figure 1:**
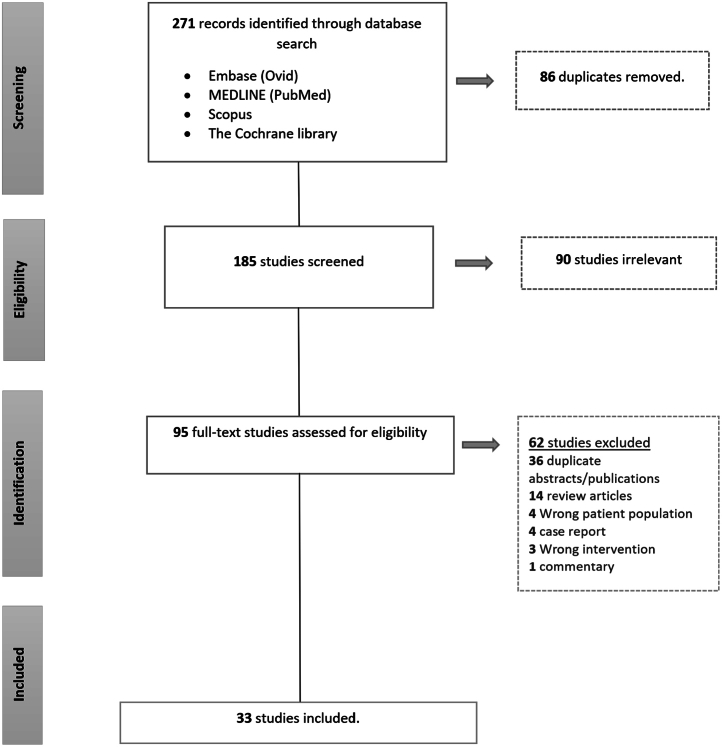
Search strategy as per preferred reporting items for systematic reviews and meta-analysis (PRISMA) guidelines.

We followed preferred reporting items for systematic reviews and meta-analysis guidelines for reporting this review (Figure 1). We performed a literature search on Embase (Ovid), Medline (PubMed), Scopus, and the Cochrane library using the keywords (‘confocal microscopy’, ‘endomicroscopy’, ‘pancreatic cyst’, ‘pancreatic cystic lesion’). There was no language or year of publication restriction. The search was performed on September 18, 2020 and yielded 271 results. We selected 33 publications and abstracts for review after removing the duplicates and excluding the review articles and irrelevant studies (Figure 1). Furthermore, any relevant study published until final submission of the article was included.

## Confocal Laser Endomicroscopy

Although CLE is a novel technique, confocal scanning microscopy was conceptualized and designed back in 1950s to visualize neuronal networks.^22^ It was not until the 90s that this technique has gained use in gastroenterology for examining intestinal epithelial proliferation.^22^^,^^23^ Since then, CLE has been investigated for use in various luminal disorders of the GI tract, including Barrett's esophagus, colorectal neoplasm, and biliary strictures, and nonluminal GI disorders like PCs.^1^

### Technical Aspects

CLE involves intravenous injection of fluorescein followed by tissue illumination by low-frequency laser light at a selected tissue depth. The fluorescence is captured through a pinhole of the endomicroscope with its probe positioned against the tissue of interest. The high-resolution images are taken within a few minutes of the dye injection.^1^^,^^24^ 'Confocal' refers to the alignment of illuminated and reflected light in the same plane so that light returning through the pinhole is selectively detected, and the scattered light beams are rejected.^2^ Thus, tissue images with high spatial resolution and architectural details are obtained.

### Types of CLE

#### Probe-Based CLE

GastroFlex, CholangioFlex, and ColoFlex are the probe-based CLE used for gastroesophageal, pancreaticobiliary, and colonic diseases, respectively. They utilize a mini probe that is introduced through the working channel of the endoscope using a catheter (Table 1). GastroFlex and ColoFlex performed during upper endoscopy and colonoscopy, respectively, are passed through the channel of standard endoscopes. The CholangioFlex probe has a smaller caliber and can be introduced during endoscopic retrograde cholangiopancreatography. Each probe has 10–20 uses. A laser scanner and a confocal processor are integrated with the probe for live image processing. Probe-based CLE has a fixed focal length and scans in a single plane.

**Table 1:** Confocal Microprobes and Their Technical Features

Types of probes	GastroFlex	AlveoFlex	CholangioFlex	AQ-Flex 19	ColoFlex
Compatible operating channel	≥2.8 mm	≥1.9 mm	≥1.0 mm	≥0.9 mm	≥2.8 mm
Length	3 m	3 m	4 m	3 m	4 m
Resolution	1 μm	3.5 μm	3.5 μm	3.5 μm	1 μm
Field of view	240 μm	600 μm	325 μm	325 μm	240 μm
Observation depth	50–55 μm	0–50 μm	40–70 μm	40–70 μm	55–65 μm

#### Needle-Based CLE

Although probe-based CLE works well for the luminal structures, the access is limited in extraluminal tissue within the pancreas. A needle-based probe was developed for CLE (nCLE) of PCs and masses.^25^ nCLE utilizes a miniprobe, AQ flex miniprobe, that is used with EUS and is loaded on a 19-gauge (g) needle. A fluorescent dye is injected intravenously followed immediately by the acquisition of endomicroscopic images. After identification of the target cyst via EUS, the needle with the probe is used to puncture the cyst with gentle engagement. The probe is advanced slightly beyond the needle tip which provides a blunt end (the probe) to contact the wall of the PC to generate images for interpretation by the endoscopist. This is repeated multiple times to obtain images at various angles. Maneuvering of the needle is minimized to lower the risk of pancreatitis. After the acquisition of the images, the probe is withdrawn. Still and movie images can be captured and saved for future review. At this point, the cyst can be aspirated for cytology and molecular analysis. The needle is then removed from the cyst.

### CLE in Luminal Disorders

The characteristic findings of Barrett's esophagus with dysplasia in CLE include dark spots and irregular structures that correspond to goblet cells and distorted capillaries, respectively.^26^ A prospective randomized study demonstrated improved sensitivity of probe-based CLE compared with white light endoscopy alone in high-grade dysplasia and early esophageal adenocarcinoma.^6^ CLE with targeted biopsies can improve the diagnostic yield of Barrett's esophagus with fewer biopsies than random biopsies.^4^ However, CLE did not improve the detection of residual lesions after endoscopic treatment of dysplastic esophageal mucosa, as shown in a multicenter randomized trial.^27^

Probe-based CLE has improved sensitivity in detecting colonic polyps compared with chromoendoscopy and narrow-band imaging. Moreover, it can increase the accuracy of detection of small polyps, thus potentially reducing the need for histopathology.^3^^,^^28^ Another area among colonic diseases where CLE has a potential role is detecting intraepithelial neoplasm in patients with inflammatory bowel disease; however, the results are conflicting without the clear advantage of CLE in improving the diagnostic yield.^5^ Intestinal metaplasia in gastric mucosa demonstrates dark spots also seen with Barrett's mucosa. Irregular capillaries, in addition to the dark disorganized features seen on CLE, are characteristic of dysplasia and can predict high-grade dysplasia or early gastric cancer with a sensitivity of approximately 90% and close to 99% specificity and accuracy.^29^ CLE has also demonstrated improvement in the diagnosis of indeterminate biliary strictures with high sensitivity and negative predictive value approaching 100% with >90% accuracy.^30^^,^^31^

### CLE in Nonluminal Disorders

nCLE utilizes a needle probe during EUS-FNA or fine needle biopsy to obtain real-time images of the inner wall of the cyst. The commonly used fine needle biopsy needle is 19-gauge. The role of CLE in PC is the characterization and subclassification of various pancreatic cystic lesions so that their malignant potential is determined accurately to guide surveillance and definitive therapy. Cross-sectional imaging (CT scans and MRI) characteristics of PC are not specific for individual cyst types. These traditional radiologic modalities can misclassify a lesion, leading to unnecessary morbidity of surgical intervention or provide a false reassurance with the future discovery of invasive pancreatic cancer.^5^, ^6^, ^7^, ^8^, ^9^, ^10^, ^11^ Thus, there is a need for a test that can differentiate PC types to facilitate a cost-effective surveillance strategy. To reliably depend on this novel technique for such critical decisions, it must be reproducible with minimum interobserver and intraobserver variability.

Distinctive features on nCLE can aid in the classification of PC types with high sensitivity and accuracy. In a retrospective analysis, nCLE significantly increased the change in management (by 43%), discontinuation of surveillance (by 32%, *P* < .05), and referral for surgery (by 10%).^32^ Prospective studies have shown substantial to complete interobserver agreement for serous cystadenoma (SCA) and pseudocysts and less optimal agreement for discriminating mucinous cyst types (IPMN and mucinous cystic neoplasm [MCN]).^33^^,^^34^ However, differentiation between mucinous and nonmucinous lesions has been achieved with confidence.^33^^,^^34^ This narrows the number of patients who require surgery and surveillance and can remarkably reduce the cost of care. INSPECT was a pilot study that assessed the safety of nCLE and practicality in differentiating PC subtypes. The sensitivity and specificity were 59% and 100%, respectively. Nine percent of patients experienced mild to moderate adverse effects, including acute pancreatitis and abdominal pain.^7^ This study raised questions about the sensitivity of the test; however, subsequent studies demonstrated optimal sensitivity arguing that there is a learning curve and need to maintain proficiency and experience as an operator to apply nCLE to clinical practice.

Krishna et al demonstrated an excellent correlation between in vivo nCLE images with ex-vivo histopathologic findings in PC. They also defined the characteristic nCLE features of individual cyst types. However, the sample size was small (N = 10) for each cyst type, limited to 2–3.^35^ A multicenter, prospective validation study involving 71 patients with conclusive nCLE (91%) provided high sensitivity, specificity, and accuracy of 95%, 100%, and 97%–99%, respectively, for the diagnosis of mucinous lesions and serous cystadenomas. The primary endpoint of the area under the curve for all types of lesions was approximately 0.98. The procedure was well tolerated, with one patient experiencing acute pancreatitis and one had nonserious bleeding in the cyst.^9^

A single-center prospective study evaluated 65 patients for the accuracy of nCLE compared with cytology or CEA with surgical histopathology as the reference. The accuracy for nCLE for mucinous cysts was significantly higher than cytology or CEA (97% [95% confidence interval {CI} 89%–100%] vs 71% [95% CI 58%–81%]). Furthermore, SCA was diagnosed with an accuracy of 97%. Moreover, it distinguished the PC subtypes (SCA, cystic neuroendocrine tumors [NET], and solid pseudopapillary neoplasm) with utmost precision with 100% sensitivity, specificity, and accuracy.^8^ In a large retrospective study (N = 206), nCLE significantly improved differentiation of indeterminate mucinous cysts into branch duct IPMN (*P* = .002) and MCN (*P* = .001). Although a significant number of patients avoided surveillance or surgery, there was no difference in the number of proposed surgeries.^34^

Probe-based CLE has been used in the diagnosis of liver nodules and to correlate with pathology with high sensitivity and specificity.^36^ Other solid intra-abdominal structures including the peritoneum, lymph nodes, and adrenal glands have been evaluated with CLE during oncologic surgery.^37^

### Confocal Endomicroscopy for Pancreatic Cysts

PC can be broadly classified based on the presence of mucin, communication with the pancreatic duct (PD), and its malignant potential. Mucinous lesions include IPMN and mucinous cystic adenomas. Most of the nonmucinous cysts are serous cystadenomas and pseudocysts and rarely cystic NET and solid pseudopapillary tumors. IPMNs arise from the main or branch PD and have a predilection for the head of the pancreas. The vast majority are main duct IPMNs. Cystic fluid stains may be positive for mucin and usually have high CEA and amylase content. Communication with the main PD (main duct IPMN) renders a high malignant potential. Other high-risk features of PC include the presence of a solid core, mural nodule, and dilatation of the main PD. MCNs arise more often in the pancreatic body or tail and lack communication with the PD. Low cyst fluid amylase can differentiate them from IPMN. They are precancerous with the highest malignant potential being in those with peripheral “eggshell” calcification.

Serous cystic neoplasms are benign nonmucinous cysts more commonly located in the body or tail of the pancreas and lack communication with the PD. The cyst fluid is low in CEA and amylase. Cystic NETs of the pancreas account for less than 1% of all PC (10%–17% of pancreatic NETs). They are usually located in the tail of the pancreas and do not contain CEA or amylase. Solid pseudopapillary tumors are the least common PC and can arise in any part of the pancreas. They are characterized by mixed solid and cystic components and low CEA and amylase. Although indolent in nature, they have malignant potential and rarely metastasize. Pseudocysts are inflammatory cysts, a consequence of pancreatitis, and can be found in any part of the pancreas. They lack mucin, have high amylase content, and have low CEA.

### Endomicroscopic Features of Individual Cyst Types

Three patterns on nCLE differentiate most of the PC types with high specificity:^33^

**Figure 2:**
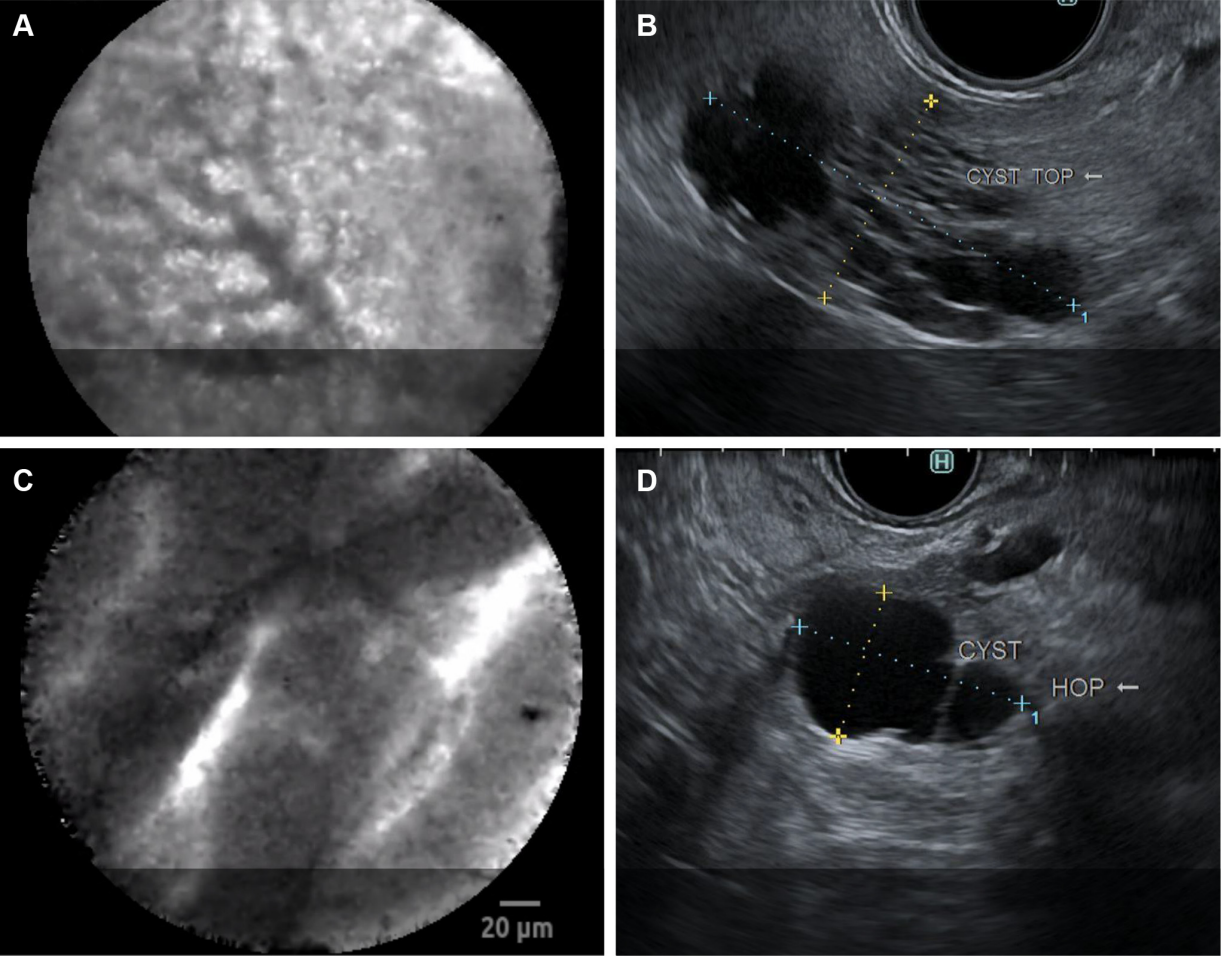
CLE and EUS images of pancreatic cysts. (A) The CLE image of serous cystadenoma with “fern pattern” of vascularity. (B) EUS of the above serous cystadenoma demonstrating cystic lesion composed of microcystic and macrocystic components. (C) CLE finding of the IPMN with papillary finger-like projections. (D) EUS of an IPMN showing anechoic cystic structure with one internal septation.
• The trabecular pattern identifies cystic NET with substantial reproducibility (Fleiss *k* value 0.78).
• The fern pattern of vascularity is distinctive of SCA (Figure 2A).

• Epithelial features with papillae and epithelial bands are characteristic of mucinous cysts (Figure 2D) with an almost complete interobserver agreement (Fleiss *k* value 0.81).

## Current Society Guidelines for the Diagnosis and Surveillance of PC

Treatment and surveillance strategy for PC endorses identification of premalignant cysts (IPMN and MCN) followed by the characterizing of high-risk features to guide further management. Characterizing PC is often challenging, reflected in current guidelines where the approach bases on “suspected” cyst type. The guidelines recommend MRI or EUS for this purpose which classifies a significant proportion of PC as undefined. CLE can classify mucinous PC with sensitivity, specificity, and accuracy exceeding 90%.^8^ Current guidelines do not endorse routine use of CLE for the diagnosis and classification of PC because of a lack of large-scale prospective studies and need for potential updates to their recommendations. Table 2 presents sensitivity, specificity, and accuracy of CT, MRI, EUS ± FNA cytopathology, CEA, and CA 19-9 in differentiating cyst type.

**Table 2:** Major Gastrointestinal Society Guidelines for the Management and Surveillance of Pancreatic Cysts

Society	Year updated	Indications for considering surgery	High-risk features for further testing	Recommended test if high-risk feature	Surveillance
ACG guidelines	2019	Symptoms (jaundice) attributed to PC.	• Size >3 cm	EUS with FNA	• IPMN and MCN: EUS or MRI at 6 mo to 2-y intervals depending on the cyst size.
		A solid component within the cyst	• Rapid growth, >3 mm/y		• Asymptomatic nonmucinous cysts: No follow-up needed
			• Main PD diameter >5 mm		
European guidelines	2018	Absolute: jaundice, main PD ≥10 mm, mural nodule ≥5 mm or a solid content within the cyst.	NA	NA	• IPMN and MCN^b^: 6-mo follow-up with MRI or EUS
		Relative: acute pancreatitis, main PD 5–10 mm, mural nodule <5 mm, cyst size ≥4 cm.			• Undefined cysts >15 mm: annual follow-up.
					• Undefined cysts <15 mm: annual follow-up for 3 y, then every other year.
					• SCN: Follow-up for 1 y.
Revised Fukuoka guidelines	2017	• Surgically fit patients with high-risk features.	“Worrisome features”	EUS for patients with “worrisome features”	If no “worrisome features” or “high-risk features”: EUS or MRI.
			• Size >3 cm,		Consideration for surgery in younger patients with cyst size >2–3 cm.^a^
			• MPD 5–9 mm,		
			• Mural nodule <5 mm,		
			• Thickened/enhanced cyst wall,		
			• Lymphadenopathy,		
			• Elevated serum CA 19-9 or		
			• Rapid growth of cyst >5 mm in 2 y.		
			“High-risk features”		
			• Jaundice in the presence of a cyst in the head of the pancreas,		
			• Main PD >10 mm or mural nodule ≥5 mm.		
AGA guidelines	2015	• Solid component and	• Size >3 cm	EUS and FNA	One high-risk feature without concerning EUS feature: Annual MRI followed by every other year.
		• Dilated main PD ± concerning EUS feature	• Dilated main PD		No high-risk feature: Annual MRI for 5 y
			• Solid component		

ACG, American College of Gastroenterology; AGA, American Gastroenterology Association; NA, not available.

a To avoid prolonged follow-up.

b Without absolute or relative indication for surgery.

## Safety of EUS-Guided Confocal Microscopy

Use of a 19-gauge needle with a diameter of greater than 1 mm (1.07 mm) to puncture the cyst during EUS-guided nCLE potentiates the risk of local complications. Adverse events have been reported in 3%–9% of procedures, the most common being acute pancreatitis, less commonly, intracystic bleeding, transient abdominal pain, and rarely cyst infection.^7^^,^^9^^,^^38^ None of the adverse events have been reported as severe. Postprocedure pancreatitis episodes are usually mild and reported in approximately 3% (1.3%–6.6%) of patients undergoing EUS-nCLE.^7^^,^^8^^,^^24^^,^^39^ Krishna et al reported 5 cases (3.5% of 144) of mild acute pancreatitis, and 4 of the 5 events of acute pancreatitis occurred during the first 25 nCLE procedures. This indicates that there is a learning curve with reduced rate of adverse events with experience. However, occurrence of acute pancreatitis had no relation with the mean duration of the procedure. Furthermore, all the episodes of acute pancreatitis occurred with the transgastric approach of needle puncture.^8^ In the largest multicenter, prospective study from France, Napolean et al reported a lower rate of postprocedure pancreatitis (1.3%). The difference in the rate of postprocedure pancreatitis could be possibly accounted by the endosonographer's experience. Adverse events associated with nCLE for PC are listed in Table 3. The overall rate of adverse events reported for EUS-FNA is approximately 5% (95% CI 1.84%–3.62%), and the rate of acute pancreatitis is about 2.0% (95% CI 0.55%–3.81%) which is comparable with that of EUS-nCLE.

**Table 3:** Adverse Events Associated With Confocal Laser Endomicroscopy for Pancreatic Cysts

Author, year	Study design	N	Adverse effect; N (%)
Krishna et al, 2020^8^	Prospective	144	Acute pancreatitis; 5 (3.5%)
Napolean et al, 2019^9^	Multicenter, prospective	206	Acute pancreatitis; 2 (1.3%)
Keegan et al, 2019^38^	Retrospective cohort	100	Acute pancreatitis; 2 (2%) Infected cyst; 1 (1%)
Nakai et al, 2015^39^	Prospective feasibility	30	Acute pancreatitis; 2 (6.6%)
Napolean et al, 2015^24^	Multicenter, prospective	31	Acute pancreatitis; 1 (3.2%)
Konda et al, 2013^7^	Multicenter, pilot	66	Acute pancreatitis; 2 (3.0%) Intracystic bleeding, 2 (3%) Transient abdominal pain, 1 (1.5%)

N, number of patients.

## Does nCLE Impact Management Decisions for PC?

The combination of nCLE with EUS-FNA provides an almost perfect diagnostic test for PC with sensitivity, specificity, and positive and negative predictive values approaching 100%.^40^ Two retrospective studies have looked at the impact of nCLE in the management of PC. Both studied solitary cysts, and the overall change in management of patients was 28% and 43%. Surveillance rates fell significantly by 35%–38%, and the rate of SCA surveillance reduced from 40% to 5%.^32^^,^^34^

## Is There Any Other Endoscopic Modality to Complement nCLE?

### EUS With Microforceps Biopsy

A pair of Moray microforceps biopsy (MFB) is introduced through the 19-gauge FNA needle to obtain a biopsy from the PC wall and mural nodule. MFB has been shown to improve the diagnostic yield in combination with EUS-FNA. The rate of tissue acquisition with MFB is approximately 90% with a diagnostic accuracy of 68%–75%.^32^^,^^41^ A systematic review (mostly including retrospective studies) comprising a pooled analysis of over 500 patients showed improvement in diagnostic yield (odds ratio 4.79, *P* = .007) compared with FNA cytology.^42^ In a retrospective analysis comparing the addition of MFB to FNA and nCLE, the use of MFB and nCLE led to discontinuation of surveillance (in 11%) and avoidance of surgery (in 25%).^32^ The diagnostic yield of nCLE was the highest (84%) and improved (93%) in combination with MFB and cytology; however, the improvement in the diagnostic yield was statistically insignificant raising questions on the value of adding MFB in setting of nCLE and FNA.^32^

Adverse events were reported in 8%–9% of patients undergoing MFB.^41^^,^^42^ Most common adverse events were mild acute pancreatitis and intracystic bleeding which were managed conservatively. The serious adverse event was rare, with approximately 1% of patients reporting severe acute pancreatitis.^42^ Although MFB has the potential to be a candidate to complement nCLE in the diagnosis of PC, the advantage of the procedure over nCLE alone is yet to be confirmed in prospective studies.

## Role of Artificial Intelligence

Artificial intelligence (AI) has the potential to ease the complexity of image interpretation in CLE and probably overcome the interobserver and intraobserver variability that exists. However, there are limited data on the utilization of AI in nCLE for PC. In a post hoc analysis of a single-center prospective study, Machicado et al^43^ examined nCLE videos of 35 patients with pathologically confirmed IPMN to develop computer-aided diagnosis algorithms. The algorithms improved the sensitivity (83% vs 56%) and accuracy (83% vs 68%–74%) for the detection of high-grade dysplasia and adenocarcinoma in IPMN compared with both the Fukuoka and American Gastroenterology Association guidelines. Nonetheless, these results are yet to be replicated in other prospective studies. There are upcoming efforts to collect data and incorporate AI to improve the performance and reproducibility of probe-based CLE. New generations of the processor and software aim to facilitate these advances in near future.

## Challenges and Limitations

### Learning Curve

As with any novel technique, there is a learning curve with CLE. This includes familiarizing with the device, understanding how to generate high-quality images, and the recognizing and interpreting characteristic CLE images of PC subtypes (mucinous and nonmucinous). Competency in the CLE techniques is being attained through continuing medical education and advanced endoscopy fellowships. There is likely variation in learning curves for various applications of CLE. A prospective, double-blind review of probe-based CLE images of colorectal neoplasms concluded that the technique and image interpretation of images are learned promptly. The training was provided with 20 CLE images of known colorectal neoplasms, followed by an assessment of obtaining high-quality images. The accuracy of image acquisition improved with the increasing number of lesions examined and plateaued over 61–76 lesions (accuracy of 86%).^43^ These data provide limited yet good evidence that endoscopists can rapidly familiarize themselves with the technique of pCLE and interpret the images with high accuracy through training and supervision. How these correlate to nCLE in PC evaluation is unknown? Studies are underway to better assess the learning curve for nCLE in PC; at this time, the use of nCLE for PCs is limited to highly trained endosonographers at academic centers, and the importance of specialized training cannot be overemphasized.

### Interobserver Agreement and Intraobserver Reliability

The technical success of nCLE has been reported to be greater than 90% in most of the published studies.^32^^,^^39^^,^^44^ However, there appears to be inconclusive evidence for the reliability and interobserver agreement on imaging features of nCLE for diagnosing PC. The interobserver and intraobserver agreement for recognizing nCLE features of SCA has been reported as nearly perfect (k 0.83, 95% CI 0.71–0.91).^33^ Likewise, the interobserver agreement for mucinous cysts was substantial (k 0.72, 95% CI 0.52–0.87) in a study of 6 unblinded investigators.^45^ However, when 15 nCLE videos were reviewed by 6 endoscopists at different centers, the accuracy between observers was 46% (range 20%–67%).^46^

Intraobserver reliability for interpreting CLE images appears to be higher among experienced observers.^47^ A retrospective analysis of institutional EUS database demonstrated high intraobserver reliability in recognizing nCLE patterns of PC among 6 blinded international endosonographers. They reviewed 29 EUS nCLE videos in 2 phases at a 2-week interval to allow washout. The intraobserver reliability for recognizing epithelial patterns ranged from 0.82 (for trabecular pattern) to 0.91 (for papillae and epithelial bands) and 0.85 for fern pattern of vascularity.^33^ This supports the need for adequate training and maintenance of proficiency when utilizing this advanced modality for clinical practice in PC evaluation.

### Other Limitations

The nCLE can provide in vivo microscopic images of the cyst wall; however, further study of the cyst is limited by the lack of a sample for molecular analysis. This limitation can be overcome by combining FNA or MFB with EUS and nCLE. It is important to note that CLE images are limited to the mucosal layer and are unable to provide information on deeper layers. The inability to examine the deep layers can be crucial in cysts with a mural nodule or solid component where biopsy may be needed. In these cases, nCLE may have specific limitations which require further study.

## Conclusions

nCLE provides real-time microscopic visualization of PC and can be a promising endoscopic modality for the diagnosis and risk stratification of these lesions. The proof of principle of this novel approach has been demonstrated in some early studies with notable follow-up studies supporting the use of nCLE in PCs. The next steps will be to validate these early results in large prospective trials. There remain challenges to overcome before widespread adoption of the technology. This includes familiarization and training with the technology among endoscopists beyond tertiary academic centers with large PC programs and need to overcome interobserver variability and cost. Furthermore, early evidence suggests a potential role of AI in nCLE to diagnose PC whereby it can enhance accuracy and support AI standardization of the process of real-time image interpretation. Looking forward, improvement in endomicroscopic technology with higher-quality images, improved proficiency with the technology, additional standardization of image patterns with AI supporting the ability to further differentiate mucinous cysts can support the applicability of nCLE as a safe and acceptable diagnostic modality which may someday become a standard of care in select PCs.
